# Identification of plasma microRNA profiles for primary resistance to EGFR-TKIs in advanced non-small cell lung cancer (NSCLC) patients with EGFR activating mutation

**DOI:** 10.1186/s13045-015-0210-9

**Published:** 2015-11-12

**Authors:** Shuhang Wang, Xiaomei Su, Hua Bai, Jun Zhao, Jianchun Duan, Tongtong An, Minglei Zhuo, Zhijie Wang, Meina Wu, Zhenxiang Li, Jian Zhu, Jie Wang

**Affiliations:** The Key Laboratory of Carcinogenesis and Translational Research (Ministry of Education), Beijing, China; Department of Thoracic Medical Oncology, Peking University School of Oncology, Beijing Cancer Hospital and Institute, 100036 Beijing, China; Department of Bioscience and Nutrition, Novum, Karolinska Institute, 141 83 Huddinge, Sweden

**Keywords:** EGFR-TKI, EGFR 19 deletion mutation, microRNA and primary resistance

## Abstract

**Background:**

*EGFR* mutation is a strong predictor of efficacy of epidermal growth factor receptor tyrosine kinase inhibitor (EGFR-TKIs) therapy in advanced non-small cell lung cancer (NSCLC). However, around 20–30 % of *EGFR*-mutated cases showed no response to EGFR-TKIs, suggesting that other determinants beyond *EGFR* mutation likely exist. This study analyzed the role of microRNAs (miRNAs) in primary resistance to EGFR-TKIs in advanced NSCLC patients with *EGFR* mutation.

**Methods:**

Training group: 20 advanced NSCLC patients with *EGFR* 19 deletion treated with first-line EGFR-TKIs were enrolled; half of them had dramatic responses while the other half had primary resistance. Matched plasma samples were collected for miRNA profiling using TaqMan low-density array (TLDA). Bioinformatics analyses were used to identify related miRNAs possibly accounted for resistance. Testing group: Quantitative reverse transcriptase PCR (qRT-PCR) was employed to detect the level of miRNA with significant differential expression in the training set. Validation group: Another cohort with *EGFR* 19 deletion mutations, who had dramatically different responses to EGFR-TKI, was used to validate the difference of miRNA expression between the sensitive and resistant groups using RT-PCR.

**Results:**

Training group: 153 miRNAs were found to be differentially expressed between the sensitive and resistant groups. Potential target genes were predicted with a target scan database. Twelve differentially expressed miRNAs were selected for the analysis because of their known roles in tumorigenesis of lung cancer, resistance to drugs, and regulation of EGFR pathway. Training group: three out of the 12 miRNAs (miR-21, AmiR-27a, and miR-218) were verified to have significantly higher expression (*P*_miR-21 = 0.004_, *P*_miR-27a = 0.009_, *P*_miR-218 = 0.041_, respectively) in the resistant group compared to the sensitive group. Validation group: The expression levels of these three miRNAs were validated to be significantly different (*P* = 0.011, 0.011, 0.026, respectively) in the validation cohort (*n* = 34).

**Conclusions:**

Higher expression levels of miR-21, AmiR-27a, and miR-218 detected in this study suggest potential roles of these miRNAs in primary resistance to EGFR-TKI in advanced NSCLC patients with *EGFR* exon 19 deletion mutations. These findings need to be further confirmed in a study with a larger sample size.

## Background

Non-small cell lung cancer (NSCLC) is a leading cause of cancer-related death. Systemic chemotherapy remains palliative and modestly effective. Recently, multiple prospective clinical studies have shown that *EGFR* mutation is a strong predictor of efficacy of epidermal growth factor receptor tyrosine kinase inhibitor (EGFR-TKIs) therapy in advanced NSCLC. More than 70 % of NSCLC patients carrying *EGFR* mutations achieved marked and durable responses to treatment with the EGFR-TKIs gefinitib or erlotinib [1, 2]. However, although several potential mechanisms of primary resistance, including Bim deletion polymorphism [3], KRAS mutation [4], and ALK fusion [5] have been explored in several preclinical and retrospective studies, the molecular basis of primary resistance to EGFR-TKI remains unclear.

MicroRNA is a newly defined class of small noncoding RNAs of 21–25 nucleotides in length that has recently been implicated in cancer biology, which could post-transcriptionally regulate gene expression by binding to complementary sequences in the 3′ untranslated region (3′UTR) of the target messenger RNA [6]. This could ultimately lead to repression of protein translation and down-regulation of protein expression [7]. Deregulation of microRNAs (miRNAs) is emerging as an important area of study in carcinogenesis because their regulatory capabilities can drastically influence cell physiology [8], and it was also reported to be with EMT which might be attributed to resistance to anti-tumor therapy [9, 10]. Many studies have examined miRNA expression profiles with the goal of identifying miRNA using non-invasive blood samples as biomarkers for the diagnosis of lung cancer. Most of these studies have quantified miRNAs in free-cellular cfDNA of serum [8, 11] or plasma [12–14], and a new strategy could be investigated accordingly [15]. Although all these studies have shown promising results, there are some limitations with the use of serum or plasma RNA for miRNA biomarker discovery. We proposed that miRNAs might regulate the *EGFR* gene pathway and could be a predictor of response to EGFR-TKI therapy. Genomic loss of miRNAs capable of down-regulating EGFR would be expected to enable increased EGFR expression, thereby offering a more robust target for the EGFR-TKIs.

Based on the fact that primary resistance to EGFR-TKIs exists in a portion of patients with a sensitizing mutant-type, it is critical to identify potential biomarkers that can help determine the subgroup of patients with primary resistance to EGFR-TKIs therapy. In this study, we investigated the expression profiles of miRNAs in *EGFR*-mutated NSCLC patients with either dramatic responses or primary resistance to EGFR-TKI therapy, and explored which miRNA may play a role in primary resistance to EGFR-TKIs in *EGFR*-mutant patients. Finally, we propose strategies to avoid primary resistance to EGFR-TKIs in the near future.

## Materials and methods

Patients enrolled in this study were diagnosed pathologically with NSCLC stage IIIB–IV or recurrent disease and received EGFR-TKIs treatment from April 2004 to August 2012 at the Beijing Cancer Hospital. All of the patients harboring *EGFR* 19 deletion mutations detected in both plasma and tissues received 250 mg of gefitinib or 150 mg of erlotinib daily until disease progression, intolerable toxicity, or patient refusal. None of the patients had KRAS mutation, T790M mutation, or C-MET amplification. All of the patients had bi-dimensionally measurable disease and presented an Eastern Cooperative Oncology Group (ECOG) performance status of 0 to 2. Blood samples were collected prior to EGFR-TKI treatment for biomarker analysis. All patients provided written informed consent and a separate consent was obtained for the optional provision of a tumor sample for biomarker analysis. The Institutional Ethics Committee at Beijing Cancer Hospital approved the study protocol.

### Study design

The study was designed to explore the potential role of miRNAs in primary resistance to EGFR-TKIs treatment. Tumor specimens were obtained at initial diagnosis. Clinical data were sealed during the laboratory analysis until all data were evaluated. Recorded variables included age, sex, smoking history, pathology, and ECOG performance status, stage at diagnosis, treatments, and toxicities. Outcome indicators included progression-free survival (PFS) and overall survival (OS). We defined primary resistance of EGFR-TKI as PFS ≤3 months (90 days) without any evidence of objective response while receiving EGFR-TKI [16].

Although both EGFR exon 19 deletions and 21 mutations (L858R) are sensitive aberrances which present excellent efficacy to EGFR-TKIs treatment, recently, a serial of pooled and meta-analysis comparing first-line chemotherapy in patients with EGFR mutations with first- and second-generation EGFR-TKIs showed that patients with EGFR 19 del had significantly longer overall survival time (OS) or/and PFS compared to those treated with platinum-based chemotherapy. On the contrary, the patients with L858R mutation had longer OS or/and PFS in the chemotherapy group than in EGFR-TKIs treatment group [17]. Thus, it has reached the consensus that EGFR19 del and L858R mutations are two different diseases and required different treatment strategies. We therefore speculated that primary resistant mechanism of EGFR exon 19 deletion and L858R mutant may be different. The current study focuses on EGFR 19 deletion mutant NSCLC.

Three cohorts of patients were selected randomly from our database:

- Training group: There were 20 advanced NSCLC patients with *EGFR* 19 deletion treated with first-line EGFR-TKIs, 10 of whom responded dramatically with a PFS over 13 months, while the other 10 presented primary resistance with a relatively short PFS of less than 3 months. Matched plasma was collected for miRNA profile detection using TaqMan low-density array (TLDA).
- Testing group: Real-time PCR was used to evaluate the differential expression of miRNAs identified in the training group. Bioinformatics analyses were applied to explore the possible miRNAs that could have been involved in the observed resistance.
- Validation group: The set included two cohorts of 17 patients each harboring *EGFR* 19 deletion mutations, with dramatic responses and primary resistance to EGFR-TKIs in each cohort were used to confirm the miRNAs identified in the training and testing sets. Another cohort of 48 *EGFR* wild-type patients with dramatic response (24 patients) or primary resistance to EGFR-TKI was chosen to verify reversely that identified miRNAs in the first two groups attributing to primary resistance of EGFR-mutant NSCLC patients have no relation with resistance of EGFR wild-type NSCLC patients.

### Assessments

Tumor assessments, including computed tomography scans of the chest, type-B ultrasonic inspection of bilateral cervical lymph nodes and upper abdomens, magnetic resonance imaging scans of brains, and emission computed tomography scans of bone, were performed at baseline and every 8 weeks until the investigators documented disease progression or unacceptable toxicity. The clinical responses to TKIs including complete response (CR), partial response (PR), stable disease (SD) and disease progression (PD) were determined on the basis of the Response Evaluation Criteria in Solid Tumors (RECIST 1.1) [18]. PFS was defined as the time from the beginning of TKI treatment to PD or death, and OS was defined as the time from the beginning of TKI treatment to death. An independent radiologist (Dr. Ning Wang) assessed all films and was blinded to the *EGFR* biomarker status.

### Specimen collection and DNA extraction

Each enrolled patient from our database exhibited specific *EGFR* mutation status (exon 19 deletion) consistently in both tissue and blood. All patients provided sufficient blood samples for miRNA analysis. Prior to TKI or second-line chemotherapy, 4 mL of anticoagulated venous blood from each patient was collected and placed at 4 °C for 4 h, then centrifuged at 2500 rpm for 10 min at low temperature. The plasma was aspirated to a new centrifuge tube, and the high speed centrifugation was repeated. Of the plasma, 1.5 mL was obtained, to which 15 μL of protease K (20 mg/mL) and 50ro SDS (20 %) was added to 1.5 ml of plasma. DNA was extracted using phenol/chloroform/isopentanol after a 2-h incubation in a water bath at 60 °C. DNA was precipitated in alcohol and centrifuged the next day. After salt washing, the precipitate was dissolved in TE and then preserved at −20 °C.

### EGFR activating mutation detection by denaturing high-performance liquid chromatography and amplification refractory mutation system

Specimen collection, DNA extraction, and denaturing high-performance liquid chromatography (DHPLC) were performed according to methods described in our previous publication [19]. The amplification refractory mutation system (ARMS), a more sensitive method for detecting *EGFR* activating mutation, was used to reevaluate the results detected by DHPLC [20].

### Quantification of circulating miRNAs from plasma

Total RNA was extracted from 350 μL of plasma using TRIzol LS reagent (Invitrogen) and dissolved in 10 μL of DEPC (diethylprocarbonate) water. The quantity and quality of total RNA were determined with the use of a spectrophotometer (GeneQuant pro, GE Healthcare). miRNAs were quantified by quantitative reverse transcriptase-polymerase chain reaction (qRT-PCR) TaqMan miRNA Assays (Applied Biosystems) according to the manufacturer’s instructions. Ten nomograms of total RNA were used to synthesize miRNA-specific complementary DNA (cDNA). PCR was performed in duplicates for 40 cycles using an ABI Prism 7900HT Sequence Detection System (Applied Biosystems). The Ct value was defined as the cycle number at which the fluorescence (ΔRn) exceeded the threshold. The threshold was 0.20, which was defined as the default setting. To evaluate the miRNA expression levels, PCR was carried out in duplicate, and the average of the Ct values was converted into 2−Ct. If the Ct value could not be determined because the PCR was run for up to 40 cycles and the miRNA expression level was still below the detection limit, the Ct value was treated as 40 (i.e., miRNA expression level = 2–40).

### MicroRNA microarray analysis of plasma and microRNA quantification

Total RNA was isolated using mirVana miRNA isolation kit (Ambion). For miRNA cDNA synthesis, RNA was reverse transcribed using a miRNA reverse transcription kit (Applied Biosystems) in combination with the stem-loop Megaplex primer pool (Applied Biosystems). TLDA v2.0 (Applied Biosystems) was performed on the 7900HT real-time PCR system (Applied Biosystems) according to the manufacturer’s protocol (764 small RNAs were profiled for each cDNA sample). PCR cycling conditions were as follows: 95 °C for 10 min followed by 40 cycles of 95 °C for 15 s and 60 °C for 1 min. Human U6 small RNA was used as an internal control to normalize RNA input. The data were analyzed using SDS v2.3 software. The Ct value was defined as the fractional cycle number at which the fluorescence passed the fixed threshold. The fold change was calculated using the 2-ΔΔCt method and presented as the fold-expression change in tumors and their adjacent normal tissues after normalization to the endogenous control. Significance was considered to be the *p* < 0.05 level.

### Target gene prediction

The target genes of the deregulated miRNAs were predicted with TargetScan; conserved and non-conserved targets of miRNAs were identified using TargetScan 3.1. The list of predicted targets was obtained via download from TargetScan (http://www.targetscan.org/).

Ingenuity Pathway Analysis (IPA) 3.0 (Ingenuity Systems 4) was used to analyze the list of predicted miRNAs targets. Filtering was performed to remove duplicates and to remove genes with no annotation in the Ingenuity Pathways Knowledge Base, resulting in a list of 765 network eligible genes. Each gene identifier was mapped to its corresponding gene object in the Ingenuity Pathways Knowledge Base. Networks of genes were then algorithmically generated. GO analysis was also performed using IPA.

### Quantitative PCR

To verify the accuracy of our microarray and TLDA data, we performed single qRT-PCR for representative miRNAs using TaqMan miRNA Assays (Applied Biosystems) according to the manufacturer’s instructions. Briefly, total RNA was extracted from plasma using TRIzol Reagent (Invitrogen, Carlsbad, CA, USA) and used to synthesize cDNA with gene-specific primers. Reverse transcriptase reactions contained 100 ng RNA, 50 nmol/L stem-loop RT primers, 1 × RT buffer, 0.25 mmol/L each of the dNTPs, 3.33 U/μL MultiScribe reverse transcriptase, and 0.25 U/μL RNase inhibitor. The 15-μL reactions were incubated for 30 min at 16 °C, 30 min at 42 °C, 5 min at 85 °C, and then kept at 4 °C. The cDNA products were used for the subsequent qRT-PCR analysis. The 20-μL PCR reaction included 1.33 μL RT product, 1 × TaqMan universal PCR master mix, and 1 μL primers and probe mix of the TaqMan miRNA assay kit. Reactions were incubated in a 96-well optical plate at 95 °C for 5 min, followed by 40 cycles at 95 °C for 15 s, and 60 °C for 1 min. PCR reactions were run on a StepOne Plus real-time PCR machine (Applied Biosystems), and the data were analyzed using SDS v2.3 software. Triplicate samples, validated endogenous controls, and interassay controls were used throughout these experiments. miRNA expression levels were calculated using the 2^-ΔΔCt^ method. The relative expression of the miRNA of interest corresponded to the 2^-miR value^.

### Statistical analyses

SPSS 16.0 was used to calculate the statistical significance. Wilcoxon tests were applied to compare the differences of expression for miRNA between the different response groups.

## Results

### Training process

Ten couples (20) of patients who met the enrollment criteria were included into the study as the training group. All patients had *EGFR* mutation both in tissues and in matched blood and lacked *KRAS* mutation, T790M, *ALK* fusion, and c-MET amplification. All patients received EGFR-TKI as the first-line therapy and demonstrated dramatic response or primary resistance to EGFR-TKI, including eight and six women, respectively, nine and six patients with lung adenocarcinomas, respectively, and non-smokers (*n* = 7 in both cases) with median PFS of 21 months and 1.8 months, respectively. The patients’ clinicopathological characteristics are listed in Table 1.

A total of 764 miRNAs were profiled for each cDNA sample with TLDA. Using a twofold difference in expression as the cut-off, 20 miRNAs were found to be highly expressed in the group with dramatic response, and 133 miRNAs were highly expressed in the group with primary resistance.

**Table 1:** Clinical characteristics of the Training group

	Responsive	Resistant
Gender		
Male	2	4
Female	8	6
Age		
Median	56	61
Smoking		
Yes	3	3
No	7	7
Pathology		
Ade	9	6
Non-Ade	1	3
Undefined NSCLC	0	1
Stage		
IIIB	2	3
IV	8	7
Median PFS (months)	21	1.8

### Gene ontology and Kyoto Encyclopedia of Genes and Genomes (KEGG) pathway analysis of the deregulated miRNAs

**Fig. 1:**
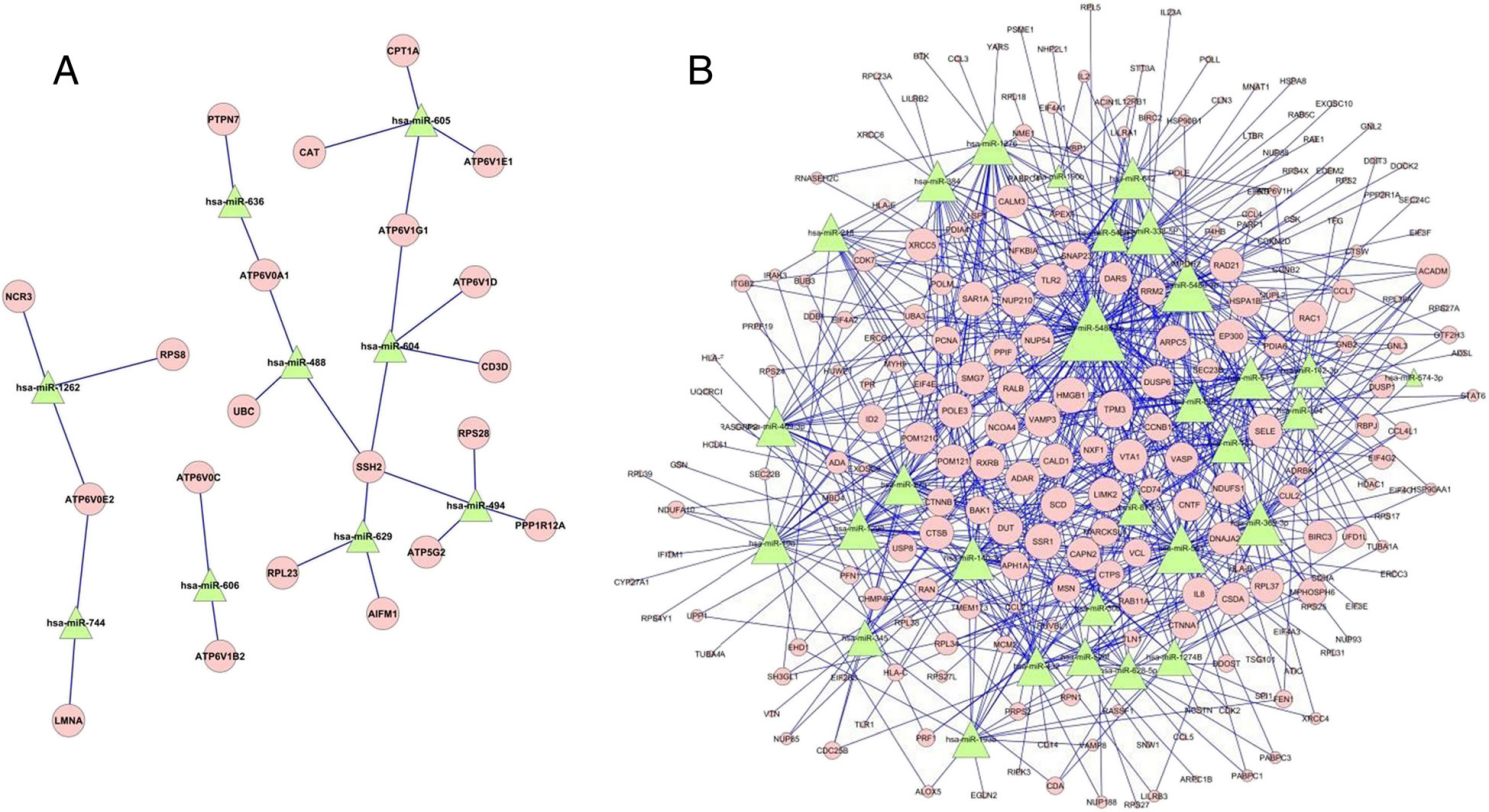
**a** miRNA up-regulated in the responsive training group and MiR-Gene Network. **b** miRNA up-regulated in the resistance training group and MiR-Gene Network. *Green nodes* represent up-regulated miRNAs; *pink nodes* represent targeted genes; and *blue lines* show the inhibitory effect of miRNAs on target mRNAs

**Fig. 2:**
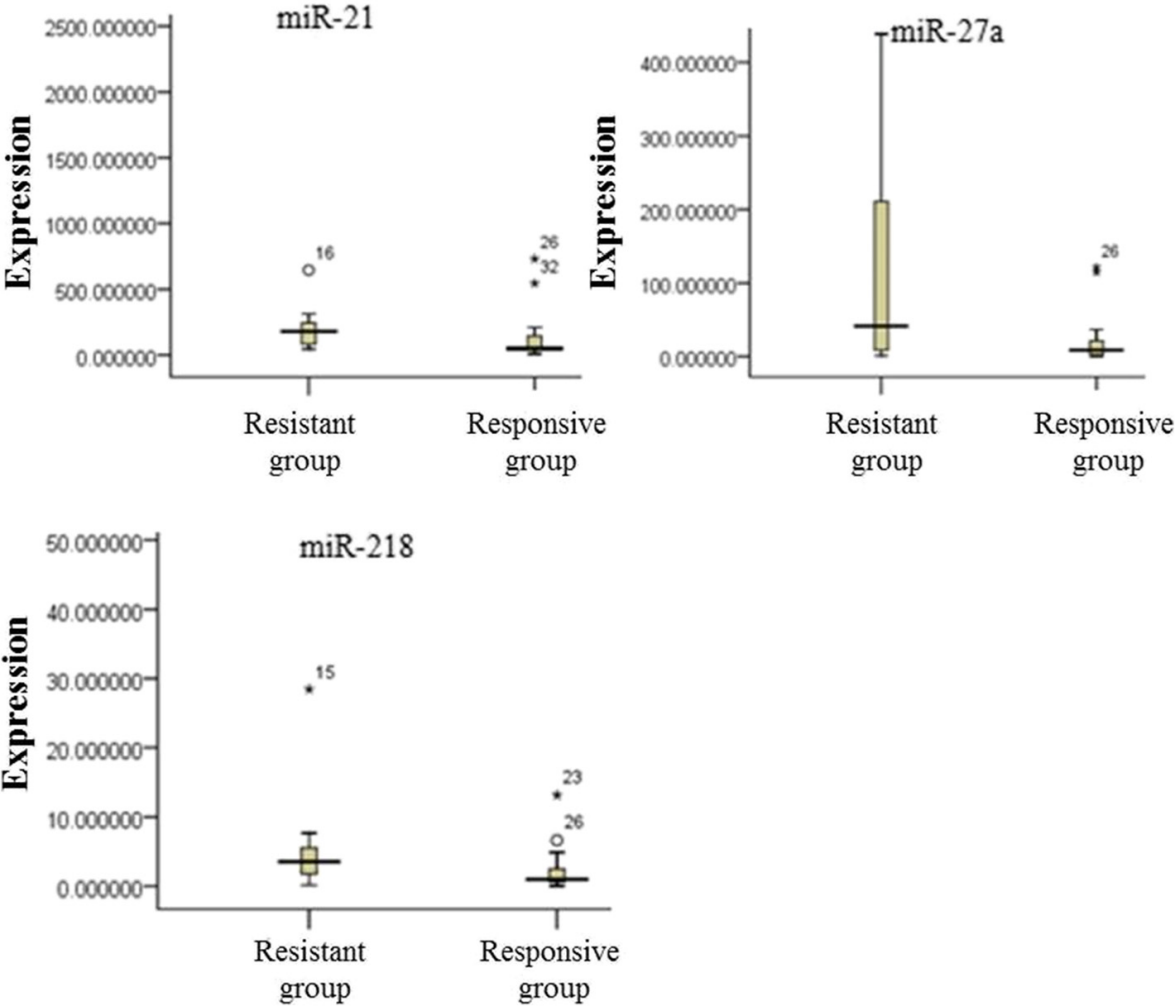
Expression level of miR-21, miR-27a, and miR-218 in the plasma of the 34 patients of the validation group

To identify the candidates and investigate the cellular function, the signaling pathway and gene ontologies (GOs) of the target genes were analyzed. The results revealed a wide variety of miRNA involved in several signaling pathways (Fig. 1a, b), such as cancer, apoptosis, and *EGFR* signaling pathway. The miRNA-mRNA interaction network analysis integrated these miRNAs and GO terms by outlining the interactions of miRNA and GO-related genes (Fig. 2a). The targets of the 12 deregulated miRNAs (Table 2) were predicted by TargetScan. miR-448 and miR-605 were upregulated in the sensitive group, whereas miR-628-5p, miR-561, miR-520f, miR-409-3p, has-miR-138, miR-296-5p, miR-218,miR-1274B, miR-21, and miR-27a were upregulated in the resistance group.

**Table 2:** Significantly differentially expressed miRNAs in the plasma of responsive and resistance groups

miRNA	Responsive group	Primary resistance group	P-value^a^
miR-21	116.50±42.55	524.38±58.27	0
miR-27a	16.16±8.09	42.03±13.18	0.01
miR-218	0.96±0.93	2.33±1.40	0.04

^a^Wilcoxon test

### Testing process

We tested the same miRNAs identified in the training group (U6 [reference miR], miR-628-5p, miR-561, miR-520f, miR-409-3p, miR-138p, miR-296-5p, miR-218, miR-1274B, miR-21, miR-27a, miR-488, and miR-605) by using qRT-PCR according to the manufacturer’s instructions. miR-21, miR-27a, and miR-218 were found to have significant differential expressions in the plasma of the sensitive and resistance groups.

### Validation process

RNAs from another 17 couples (34) of patients harboring *EGFR* 19 deletion with dramatic response or primary resistance to EGFR-TKIs therapy were evaluated by qRT-PCR analysis for the detection of miR-21, miR-27a, and miR-218. The results are presented in Table 3.

**Table 3:** Validated significantly differentially expressed miRNAs in the plasma of responsive and resistance groups in 17 couple of patients with EGFR 19 mutation

miRNA	Responsive group	Resistant group	P-value^a^
miR-21	135.98±26.44	182.91±47.63	0.01
miR-27a	22.38±5.66	131.85±53.75	0.01
miR-218	1.59±0.714	5.09±1.65	0.03

^a^Wilcoxon test (*P*<0.05)

### EGFR wild-type group

miR-21, miR-218, and miR-27a were also detected by qRT-PCR from 24 couples (48) of patients with *EGFR* wild-type who presented response (*n* = 24) or resistance (*n* = 24) to EGFR-TKIs. Patient characteristics are shown in Table 4, and no significant difference was found.

**Table 4:** Clinical characteristics of the EGFR wild type group

	Responsive	Resistance
	Group	Group
Gender		
Male	6	13
Female	18	11
Age		
Median	62	58
Smoking		
Yes	6	9
No	18	15
Pathology		
Ade	21	18
Non-ade	3	6
Stage		
IIIB	4	7
IV	20	17
Median PFS(months)	17.1(14.8-51.3)	2.1 (0.3-3.5)

## Discussion

The mechanism of primary resistance to EGFR-TKIs in *EGFR*-mutant NSCLC is not clearly understood. In this study, we analyzed miRNA expression signatures related to primary resistance to EGFR-TKIs in *EGFR*-mutant NSCLC patients. Our results indicate the existence of miRNA expression patterns that could distinguish between sensitive and resistant NSCLC patients with *EGFR* activating exon 19 deletion mutations, therefore identifying those who could benefit from EGFR-TKIs and providing promising therapeutic opportunities.

In the present study, we identified three miRNAs potentially related with primary resistance to EGFR-TKIs in exon 19-mutated NSCLC. We subsequently utilized another cohort of 19 exon-mutated patients to validate this result. Moreover, we thought it is necessary to use another subgroup of patients with wild-type EGFR who had matched short and long PFS to verify reversely that the identified miRNAs in the first two groups contribute to primary resistance of EGFR 19 del NSCLC patients, but have no relation with resistance of EGFR wild-type NSCLC patients. Forty-eight EGFR wild-type patients came from our database; we intentionally chose this subgroup of patients with the matched longest and shortest PFS. Multiple studies have suggested that a small subgroup of patients without EGFR mutation (around 10 %) also can benefit from EGFR-TKIs therapy; it might attribute to some uncommon mutations of EGFR (such as G719 and L861) [21] or other unknown mechanisms for now. What is more, in our database, we found that around 50 % of patients in this study underwent EML4-ALK detection (FISH or VATANA), and no positive cases were found. As we know, EML4-ALK fusion and EGFR mutation are often exclusive for most patients, and incidence rate of overlapping EML4-ALK fusion and EGFR sensitive mutation was only about 0–8 % in NSCLC patients [5, 22].

In our study with TaqMan miRNA array and qRT-PCR confirmation, 12 deregulated miRNAs were predicted by TargetScan in conjunction with bioinformatics methods (miR-448, miR-605, miR-628-5p, miR-561, miR-520f, miR-409-3p, has-miR-138, miR-296-5p, miR-218, miR-1274B, miR-21, and miR-27a) in a training set. Three up-regulated miRNAs (miR-21, miR-27a, and miR-218) were identified in *EGFR*-mutant NSCLC patients resistant to EGFR-TKIs in the testing and validating cohorts.

High levels of miR-21 expression have been reported in various types of human tumors including lung cancer [23–25]. However, the mechanism that up-regulates miR-21 during carcinogenesis has not been identified yet. Seike et al. reported in 2009 that miRNA microarray data showed higher levels of miR-21 in *EGFR*-mutant cases [26], and in vitro analyses using NSCLC cell lines showed that activated EGFR signaling up-regulated miR-21 expression. A statistically significant positive correlation was observed between miR-21 expression levels and p-EGFR levels in NSCLC cell lines. Furthermore, treatment with the EGFR-TKI (AG1478) inhibited miR-21 expression in two NSCLC cell lines with elevated p-EGFR, *EGFR*-mutant H3255, and *EGFR* wild-type H441, providing a mechanistic link between an activated EGFR signaling pathway and the aberrant up-regulation of miR-21, and a therapeutic basis for the inhibition of miR-21 in lung cancers with EGFR activation. Following antisense oligonucleotide-mediated knockdown of miR-21, induced or enhanced apoptotic responses in two NSCLC cell lines, H3255 and H441, suggested that miR-21 could also be a therapeutic target in resistant *EGFR*-mutant NSCLC. Recent reports suggested that miR-21 overexpression was associated with resistance to cytotoxic agents, including gemcitabine [27], docetaxel [28], temozolomide [29], and taxan [30]. A recent study [31] based on animal model and clinical samples clarified that miR-21 overexpression was associated with acquired resistance to EGFR-TKI in NSCLC, which might be caused by miR-21’s function in activating the PI3K/AKT pathway through PTEN and PDCD4 inhibition. In our study, we identified miR-21 as a potential biomarker of resistance to EGFR-TKIs in *EGFR*-mutant patients. This is the first study that identified a potential association between miR-21 and primary resistance to EGFR-TKIs in *EGFR*-mutant patients. This finding needs to be further verified with in vitro and in vivo studies of molecular mechanisms and in larger clinical studies.

Studies involving associations of miR-27a and miR-218 with resistance to EGFR-TKIs in NSCLC are extremely limited for now. T790M mutation and *MET* gene amplification have been considered as important acquired resistant mechanisms. Recently, several studies also reported that T790M mutation or *MET* gene amplification could co-exist with *EGFR* sensitive mutation in therapy-naïve patients [20, 32], suggesting that these two mechanisms may play important roles in primary resistance to EGFR-TKIs. Yoon and his colleagues [33] identified a mechanism linking miR-27a, MET, and EGFR that involves Sprouty2, and thus illustrating cross-talk between MET and EGFR in NSCLC. This study also showed that EGFR and MET receptor tyrosine kinases, through regulation of expression of specific miRNAs such as miR-218, control the metastatic behavior and gefitinib resistance of NSCLCs. Garofalo et al. [34] reported that the modulation of specific miRNAs, such as miR-30b, miR-30c, miR-221, and miR-222, might play a role in acquired resistance to EGFR-TKIs and could have therapeutic applications to sensitize lung tumors to TKI therapy [35]. However, these studies mainly focused on the association of plasma miRNA and MET in acquired resistance to EGFR-TKIs. The present study is the first to identify that miR-27a, miR-218, and miR-27a might play a role in primary resistance to EGFR-TKI in advanced NSCLC patients with *EGFR* exon 19 deletion mutations treated with EGFR-TKI, independent of c-MET amplification and T790M mutation. Other relevant mechanisms should be further investigated in basic research [36–38].

## Conclusion

In summary, increased expression of miR-21, AmiR-27a, and miR-218 may play a role in primary resistance to EGFR-TKI in advanced NSCLC patients who had EGFR exon 19 deletion mutations. This finding needs to be further confirmed in clinical study with a larger sample size. Finally, we propose that miRNAs targeted treatment combined with TKIs might provide a new strategy to treat NSCLCs in the future.

## Abbreviations

3′UTR: 3′ untranslated region
ARMS: amplification refractory mutation system
CR: complete response
DHPLC: denaturing high-performance liquid chromatography
EGFR-TKIs: epidermal growth factor receptor tyrosine kinase inhibitors
GO: gene ontology
IPA: ingenuity pathway analysis
KEGG: Kyoto encyclopedia of genes and genomes
NSCLC: non-small cell lung cancer
OS: overall survival
PDCD4: programmed cell death gene 4
PFS: progression-free survival
PR: partial response
qRT-PCR: quantitative reverse transcriptase PCR
RECIST: Response Evaluation Criteria in Solid Tumors
SD: stable disease
TLDA: TaqMan low-density array
